# 

**Published:** 1904-11

**Authors:** 


**FIVE HUNDRED EXTRA COPIES THIS ISSUE.**

Twentieth Year.

VOL. XX. NOVEMBER, 1904. No. 5.

LIBRARY
SURGEON GENERAL'S OFFICE
DEC. 1 - 1904

LIBRARY
INDEXED
S. G. O.

### TEXAS Medical Journal.

SUBSCRIPTION, $1.00 A YEAR, IN ADVANCE.

SINGLE COPIES, 10 CENTS.

PUBLISHED AT AUSTIN, TEXAS, BY
F. E. DANIEL, M. D.,
PROPRIETOR.

PRINTED BY THE VON BOECKMANN-JONES CO.

Entered at the Postoffice at Austin, Texas, as Second-class Mail Matter.

